# 33.4 UNDER WHAT CONDITIONS DO YOUNG PEOPLE DISCLOSE THEIR DIFFICULTIES? SUBJECTIVE EXPERIENCES OF YOUNG PEOPLE AT RISK OF DEVELOPING PSYCHIATRIC DISORDER

**DOI:** 10.1093/schbul/sby014.140

**Published:** 2018-04-01

**Authors:** Petra Gronholm, Graham Thornicroft, Kristin R Laurens, Sara Evans-Lacko

**Affiliations:** 1 London School of Economics and Political Science; 2 King’s College London; 3 University of New South Wales

## Abstract

**Background:**

Stigma and discrimination are proposed as critical factors contributing to the underuse of mental health services amongst young people, however these influences remain understudied. Existing research on stigma experienced by young people has focused on individuals in contact with mental health services or with a psychiatric diagnosis. Using a community sample, this study investigates subjective accounts of stigma during the early stages of mental health difficulties with regards to how disclosure and coping are considered, and how help-seeking is approached.

**Methods:**

In-depth semi-structured individual interviews were conducted with young people from a Greater London, UK, community cohort. Purposive sampling criteria were used to recruit participants who reported early psychopathology of a persisting nature (emotional and/or behavioural difficulties at a clinical level, and psychotic-like symptoms), thus representing young people at-risk of developing psychiatric disorder. 29 young people aged 12-18 years took part in the study. Thematic analysis was used to analyse the interview data.

**Results:**

In-depth semi-structured individual interviews were conducted with young people from a Greater London, UK, community cohort. Purposive sampling criteria were used to recruit participants who reported early psychopathology of a persisting nature (emotional and/or behavioural difficulties at a clinical level, and psychotic-like symptoms), thus representing young people at-risk of developing psychiatric disorder. 29 young people aged 12-18 years took part in the study. Thematic analysis was used to analyse the interview data.

**Discussion:**

“Conditional disclosure” is central to how young people cope with their difficulties. Often stigma-related concerns in particular contributed to restricted disclosure, in this way delaying young people’s initial help-seeking when difficulties emerge.

